# Peripheral blood T Regulatory cell counts may not predict transplant rejection

**DOI:** 10.1186/1471-2172-11-40

**Published:** 2010-07-15

**Authors:** Yuchuan Huang, Juan Shan, Chuntao Zhang, Jie Zhang, Li Feng, Shengfu Li, Youping Li

**Affiliations:** 1 Key Laboratory of Transplant Engineering and Immunology, Ministry of Health, West China Hospital, Sichuan University, Chengdu China; 2 Technology Research and Development Center, China Tobacco Chuanyu Industrial Corporation. Chengdu China; 3 Chinese Cochrane Centre, Chinese Evidence-Based Medicine Centre. Chengdu China

## Abstract

**Background:**

Recent evidence shows that allograft survival rates show a positive correlation with the number of circulating T regulatory cells (Tregs). This study investigated both the number and the cytokine profiles exhibited by Foxp3^+ ^Tregs in blood, spleen and lymph nodes of Lewis rat recipients of BN rat cardiac allografts after a single-dose of Rapamycin (RAPA).

**Results:**

Rats were divided into three groups: control group (containing healthy control and acute rejection group), and recipients treated with a single dose of RAPA on either Day 1 (1D group)or Day 3 (3D group) post-transplant. We analyzed the number of Foxp3+Tregs and the expression of Foxp3 and cytokines in the peripheral blood and the peripheral lymphoid tissues. No difference was found in the numbers of circulating Foxp3+ Tregs between these three groups. RAPA administration significantly increased Foxp3 expression in peripheral lymphoid tissues after a single dose of RAPA on Day 3 post-transplant. Foxp3+Tregs inhibited the activity of effector T cells (T_eff_) via the secretion of TGF-β1.

**Conclusion:**

The number of Tregs in the recipient's blood may not be a good predictor of transplant rejection. Foxp3+Tregs inhibit the activity of T_eff _cells mainly in the peripheral lymphoid tissues.

## Background

Organ transplantation remains the only solution available for the treatment of numerous end-stage diseases. The administration of immunosuppressive drugs following solid organ transplantation is essential in order to control the activation of alloreactive T cells and so prevent rejection of the graft. Although calcineurin inhibitors (CNI), anti-proliferative agents and steroids all have proven efficacy, immunosuppression after heart transplantation may still be improved, with respect to increased efficacy (reduced incidence of acute rejection and chronic allograft vasculopathy (CAV) and reduced toxicity (less renal failure and malignancy). The proliferation signal inhibitor (PSI) Rapamycin (RAPA), and its derivative everolimus, belong to a new class of potent therapeutic agents that promise such an improvement. Research has shown that delayed treatment with RAPA effectively slows the progression of both allograft rejection [1] and pre-existing CAV in both rat [2] and non-human primate grafts [3,4]. Recently, ex vivo studies have shown that, in both humans and mice, RAPA selectively blocks the proliferation of effector T cells (T_eff_) while at the same time, expanding the CD4^+^CD25^+^FoxP3^+ ^Treg population [5].

Tregs control various immune responses and play an important role in inhibiting transplant rejection [6]. It has been reported that graft survival rates are positively correlated with the number of circulating Tregs[7,8], and that the adoptive transfer of Tregs (polyclonally expanded in vivo using low-dose RAPA) promotes tolerance to allogeneic pancreatic islet grafts [9].

In a retrospective study, Segundo et al[8] found that RAPA (but not CNI) induces an increase in the number of circulating Tregs in stable renal transplant recipients. Therefore, we analyzed both the number and the cytokine expression levels in Foxp3+ Tregs in the blood, spleen and lymph nodes of rat transplant recipients after a single post-operative dose of RAPA. Our data show that Foxp3+ Tregs inhibit T_eff _cell activity in the peripheral lymphoid tissues by secreting cytokines such as TGF-β1. We also suggest that the number of circulating Tregs may not be a suitable predictor of transplant rejection.

## Methods

### Rats and heart transplantation

Donor male Brown Norway (BN, RT-1^n^) and Lewis (RT-1^1^) recipient rats weighing 150-200 g were purchased from Experimental Animal Centre of West China (Sichuan University, Chengdu, China). Rats were divided into three groups as follows: three rats were used as healthy controls; nine rats underwent cardiac allograft transplantation with no follow-up treatment (acute rejection (Con) group); and 24 rats underwent cardiac allograft transplantation followed by a single-dose of RAPA given orally either 1 day (1D group) or 3 days (3D group) post transplantation. Ethical approval for the work was given by the University Committee on Use and Care of Laboratory Animals at Sichuan University.

**Figure 1:**
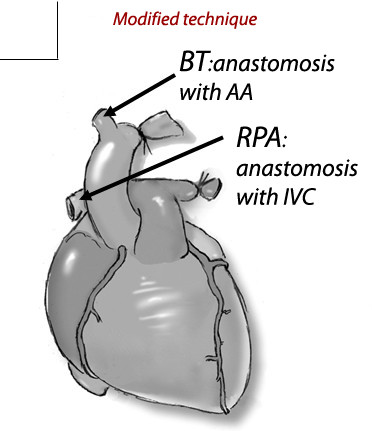
**Modified abdominal heart transplantation model in rat**. The brachiocephalic trunk (BT) and the right pulmonary artery (RPA) were anastomosed to the abdominal artery (AA) and inferior vena cava(IVC) in an end-to-side fashion, respectively.

We used a modified heart abdominal transplantation [10] and donor hearts were harvested according to the method of Ono and Lindsey [11]. Briefly, the aortic arch was ligated between the brachiocephalic trunk and the left common carotid artery. The vessel was then cut distal to the ligature. The brachiocephalic trunk was transected, leaving 2-3 mm attached to the aortic arch. The left pulmonary artery was ligated and the right pulmonary artery transected, leaving 2-3 mm attached to pulmonary artery. The abdomen was then opened by a long midline incision. The abdominal aorta was clamped proximally and distally and a 3-0 silk tie looped around both distal vessels and kept loose. The donor brachiocephalic trunk was connected end-to-side to the recipient abdominal artery. When finishing the arterial anastomosis, the silk was tied and the distal vascular clamp was moved to the proximal inferior vena cava. The donor right pulmonary artery was then connected end-to-side to the recipient inferior vena cava (Fig 1).

### Rapamycin treatment and analysis of whole blood RAPA concentrations

A single dose of Rapamycin (RAPA; 1 mg/ml, NCPC New Drug Research and Development Co., Ltd.) was given intragastrically at 8 mg/kg on either Day 1 (1D group) or Day 3 (3D group) post-transplantation. Physiological saline was given to the Con group. At least three independent experiments were performed.

Whole-blood trough RAPA concentrations were dynamically determined using a modified high-performance liquid chromatography spectrometry method [12].

### Flow Cytometry Analysis

Peripheral Blood samples were taken from the Con group 1, 3 and 6 days after transplantation and from the 1D and 3D groups 1, 3, 7 and 10 days after treatment. The percentage of CD3^+^CD25^+^Foxp3^+ ^T cells was determined using three-color flow cytometry. Briefly, lymphocytes from peripheral blood were collected and suspended in phosphate-buffered saline (PBS) and then labeled with an optimal concentration of FITC-conjugated mouse anti-rat CD3 antibody (G4.18; BD Pharmingen, California, USA) and Allophycocyanin (APC) anti-rat CD25 (OX-39; eBioscience, San Diego, USA) in the dark for 30 minutes. Intracellular staining was performed using a PE anti-mouse/rat/human Foxp3 Flow Kit (150D, BioLegend, San Diego, USA). Isotype-matched controls were run concurrently.

### Immunohistochemistry (IHC)

Hearts, spleens and lymph nodes were harvested from the Con group 1, 3 and 6 days after transplantation (POD 1, 3, 6) and from the 1D and 3D groups 1, 3, 7 and 10 days after treatment. Formalin-fixed, paraffin-embedded tissue sections (4-μm) were stained with a 1:100 dilution of affinity purified anti-muse/rat Foxp3 (FJK-16s, eBioscience) followed by incubation with the respective biotinylated secondary antibodies. Staining was developed using an avidin-biotin-based detection system and visualized with peroxidase or DAB (Dako REAL™ EnVison™ Detection System, Denmark A/S). The number of stained cells in each section was counted in 10 ocular grid areas at a magnification of ×200 and the total was divided by the number of ocular grid areas.

### RNA preparation and real-time PCR

The levels of Foxp3, IL-10, TGF-β1 and β-actin mRNA were analyzed by quantitative real-time RT-PCR using a Light Cycler (Multicolor Real-Time PCR detection system). Total RNA isolation and real-time PCR was carried out according to the manufacturer's instructions. Briefly, each 20 μl of reaction volume contained 3 mM MgCl_2_, Light Cycler Hotstart DAN SYBR Green I Mix, specific primer pairs, and 5 μl of cDNA. PCR cycling conditions were 95°C for 3 minutes followed by 45 cycles at 95°C for 15 seconds, 60°C for 15 seconds and 72°C for 15 seconds. Primers were designed based on the reported cDNA sequences; the expected fragment sizes for these genes are specified in Table 1.

**Table 1:** Primers used in this study

Gene		Primer	annealing temperature
TGF-β1	sense	5'-CTGCTGCCGCTTCTGCTC-3'	56°C
	antisense	5'-TGCGCTTCCGTTTCACC-3'	
IL-10	sense	5'-CAGACCCACATGCTCCGAGA-3'	51°C
	antisense	5'-CAAGGCTTGGCAACCCAAGTA-3'	
Foxp3	sense	5'-ACCTCCTCTTCTTCCTTGAACC-3'	56°C
	antisense	5'-GCATAAAGTGTGGTCTGTCCTG-3'	
GAPDH	sense:	5'-CGGCAAGTTCAACTTCACA-3'	56°C
	antisense	5'-AAGACGCCAGTAGACTCCACGA-3'	

### Statistical analysis

Student's t-test was used for paired and unpaired analyses. A statistical evaluation for graft survival was performed using the Kaplan-Meier test. p < 0.05 was considered statistically significant. All experimental data were representative of three independent experiments.

## Results

### Effect of delayed RAPA treatment on heart allograft survival

**Figure 2:**
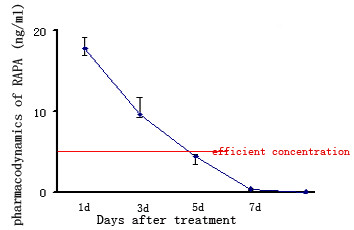
**Pharmacokinetics of RAPA in blood samples obtained from recipients**. The extractions of RAPA from blood samples were injected onto tandem supelcosil C18 columns (Supelco, Bellefonte, PA) maintained at 40°C and eluted with mobile phase buffer composed of a 90:10 mixture of methanol:water at a flow rate of 0.5 ml/minute. UV absorbance was monitored at 276 nm. We found that the concentration of RAPA was below its effective concentration (5-15 ng/ml) 5 days after treatment.

The donor heart beat strength was checked on a daily basis. Untreated Lewis rat recipients of BN heart allografts had a mean graft survival time of 5.2 ± 1.3 days. Rats given a single dose of RAPA on either Day 1 or Day 3 post transplant showed significantly prolonged graft survival times of 11 ± 1.5d (p < 0.05) or 14 ± 2d (p < 0.05), respectively. Furthermore, the graft survival rate in D3 group was significantly longer than that in D1 group (P < 0.05). We found RAPA was below its effective concentration (5-15 ng/ml) 5 days after treatment commenced (Fig 2).

### Peripheral Blood Treg Counts

**Figure 3:**
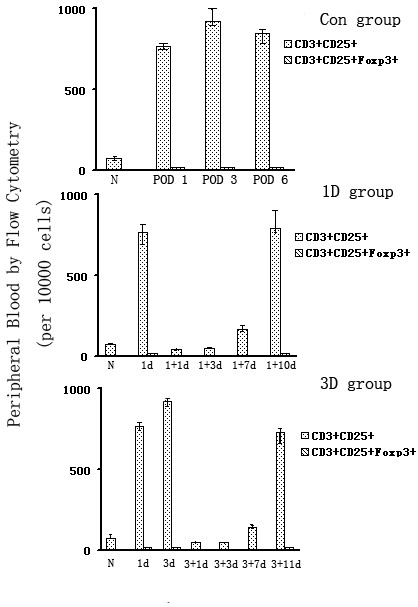
**Quantification of Tregs in the peripheral blood of recipient rats**. The number of CD3+CD25+Foxp3+ Tregs cells in the blood of rats treated with RAPA on Day 1 (1D group)or Day 3 (3 D group) post-transplant were compared. Normal subjects (N) and heart transplant recipients without any immunosuppression were used as controls (Con group). No statistically significant difference between the groups was found among these three groups. Con group N: Normal spleen; POD 1: 1^st ^day after transplantation; POD 3: 3^rd ^days after transplantation; POD6: 6^th ^day after transplantation; 1 D group 1+1d: 1^st ^day after RAPA treatment in 1D group; 1+3d: 3^rd ^day after RAPA treatment in 1D group; 1+7d: 7^th ^day after RAPA treatment in 1D group; 1+10d: 10^th ^day after RAPA treatment in 1D group; 3D group 3+1d: 1^st ^day after RAPA treatment in 3D group; 3+3d: 3^rd ^day after RAPA treatment in 3D group; 3+7d: 7^th ^day after RAPA treatment in 3D group; 3+11d: 11^th ^day after RAPA treatment in 3D group.

**Figure 4:**
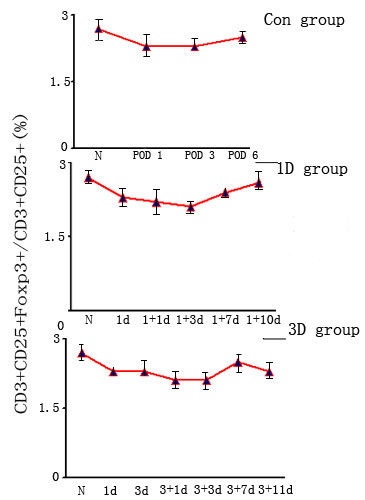
**The rates of CD3+CD25+Foxp3+/CD3+CD25+ in the peripheral blood of recipient rats**. The rates of CD3+CD25+Foxp3+/CD3+CD25+ in the blood of rats treated with RAPA on Day 1 (1D group)or Day 3 (3 D group) post-transplant were compared. Normal subjects (N) and heart transplant recipients without any immunosuppression were used as controls (Con group). No statistically significant difference between the groups was found among these three groups. Con group N: Normal spleen; POD 1: 1^st ^day after transplantation; POD 3: 3^rd ^days after transplantation; POD6: 6^th ^day after transplantation; 1 D group 1+1d: 1^st ^day after RAPA treatment in 1D group; 1+3d: 3^rd ^day after RAPA treatment in 1D group; 1+7d: 7^th ^day after RAPA treatment in 1D group; 1+10d: 10^th ^day after RAPA treatment in 1D group; 3D group 3+1d: 1^st ^day after RAPA treatment in 3D group; 3+3d: 3^rd ^day after RAPA treatment in 3D group; 3+7d: 7^th ^day after RAPA treatment in 3D group; 3+11d: 11^th ^day after RAPA treatment in 3D group.

We used Flow Cytometry to count the number of circulating CD3^+^CD25^+^Foxp3^+ ^Tregs. We were surprised to find no real differences in the number of circulating CD3^+^CD25^+^Foxp3^+ ^Tregs between the RAPA-treated group and the normal controls or the acute rejection group (p > 0.05) (Fig 3 and Fig 4).

### Expression levels of Foxp3 differ with time of RAPA administration post-transplant

**Figure 5:**
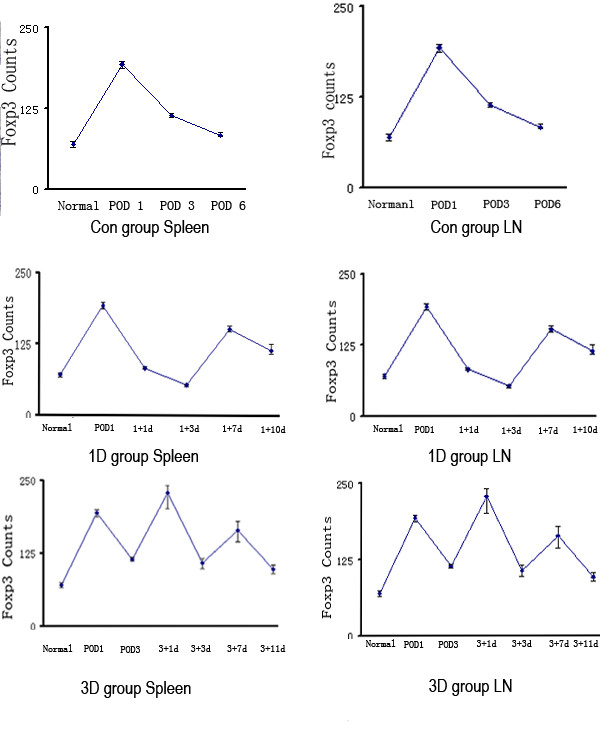
**Time course and localization of Foxp3+ Tregs in spleens and lymph nodes assessed by immunohistochemistry**. We assessed the dynamic changes in the number of Foxp3+ Tregs in the spleens by immunohistochemistry. Tissue sections were stained using an anti-Foxp3 monoclonal antibody. The positive cells in the spleens were counted in 10 ocular grid areas within each section at a magnification of ×200, and total was divided by the number of ocular grid areas. We counted no less than 6 sections in one time point. We assessed the dynamic changes in the numbers of Foxp3+ Tregs in lymph nodes (LN) by immunohistochemistry. Tissue sections were stained using an anti-Foxp3 monoclonal antibody. The positive cells in spleens were counted in 5 ocular grid areas within each section at a magnification of ×200, and total was divided by the number of ocular grid areas. We counted no less than 6 sections in one time point. Con group POD 1: 1^st ^day after transplantation; POD 3: 3^rd ^days after transplantation; POD6: 6^th ^day after transplantation; 1 D group 1+1d: 1^st ^day after RAPA treatment in 1D group; 1+3d: 3^rd ^day after RAPA treatment in 1D group; 1+7d: 7^th ^day after RAPA treatment in 1D group; 1+10d: 10^th ^day after RAPA treatment in 1D group; 3D group 3+1d: 1^st ^day after RAPA treatment in 3D group; 3+3d: 3^rd ^day after RAPA treatment in 3D group; 3+7d: 7^th ^day after RAPA treatment in 3D group; 3+11d: 11^th ^day after RAPA treatment in 3D group.

To characterize the Foxp3+ Tregs in the peripheral lymphatic organs, we analyzed Foxp3 expression using immunohistochemistry and real-time PCR. No Foxp3 expression was seen in the rejected heart allografts. The number of Foxp3+ Tregs increased by Day 1 post-transplant in the spleens compared with normal spleens. From Day 2 post-transplant, Foxp3+ Treg numbers in the spleens of the acute rejection group decreased. In the 1D group, the number of Foxp3 Treg cells in the spleens decreased on Days 1-3, increased on Days 3-7 and then decreased again. However, the 3D group showed a different pattern. Foxp3 Treg cell numbers showed a peak on Day 1, decreased on Days 1-3, and peaked again on Day 7 before decreasing again. It is interesting that the time of RAPA administration post-transplant has different effects upon Foxp3 expression by the Tregs. It is also noteworthy that Foxp3 expression by the Tregs was higher in the lymph nodes than in the spleens. (Fig 5, additional file 1 and 2)

### Treg suppression of T_eff _cell activities

**Figure 6:**
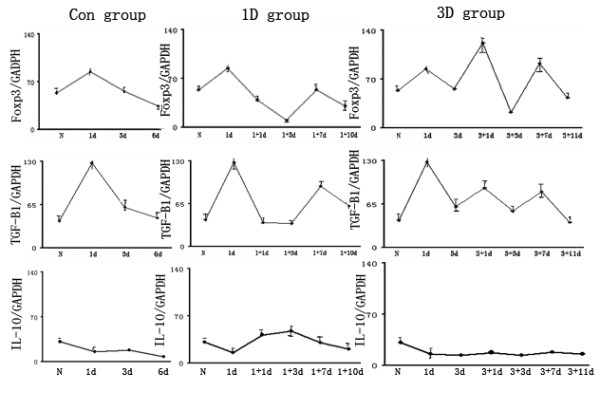
**The expression of Foxp3, TGF-β1 and IL-10 mRNA in spleens assessed by real-time PCR**. Cytokines are known to play an important role in immunological regulation by Tregs. Therefore, we measured mRNA levels for the inhibitory cytokines, TGF-β1 and IL-10. We showed that the dynamic expression profile of TGF-β1 mRNA was the same as that of Foxp3 mRNA. This was not the case for IL-10 mRNA. Con group N: Normal spleen; POD 1: 1^st ^day after transplantation; POD 3: 3^rd ^days after transplantation; POD6: 6^th ^day after transplantation; 1 D group 1+1d: 1^st ^day after RAPA treatment in 1D group; 1+3d: 3^rd ^day after RAPA treatment in 1D group; 1+7d: 7^th ^day after RAPA treatment in 1D group; 1+10d: 10^th ^day after RAPA treatment in 1D group; 3D group 3+1d: 1^st ^day after RAPA treatment in 3D group; 3+3d: 3^rd ^day after RAPA treatment in 3D group; 3+7d: 7^th ^day after RAPA treatment in 3D group; 3+11d: 11^th ^day after RAPA treatment in 3D group.

Tregs are fundamental in controlling various immune responses and many different subsets of Tregs have been reported. Among these subsets are the induced Tregs (iTregs) that produce high levels of IL-10 and inhibit T_eff _cells[13]. Foxp3^+ ^Tregs also inhibit T_eff _cells by secreting TGF-β1/IL-10 [13]. To assess the role of IL-10 and TGF-β1 in our experiments, real-time PCR was used to examine the expression of both IL-10 and TGF-β1 mRNA in recipient Lewis rat spleens. We found that the expression pattern of TGF-β1 mRNA was the same as that of Foxp3 mRNA (Fig 6). Both displayed a peak on Day 1, decreased on Days 1-3, and peaked again on Day 7 before decreasing again. However, this was not the case for IL-10 mRNA.

## Discussion

Recently, it has been shown that RAPA selectively blocks the proliferation of effector T cells while at the same time expanding the population of both human and mouse CD4^+^CD25^+^FoxP3^+ ^Tregs ex vivo [1]. In patients transplanted with heart [14], lung [15], liver [7] or kidney [8] grafts, graft survival rates showed a positive correlation with the number of circulating Tregs. Therefore, in this study, we monitored the number of circulating CD3^+^CD25^+^Foxp3^+ ^Tregs in the peripheral blood of rats transplanted with cardiac allografts. We show that RAPA did not affect the number of circulating Tregs when compared with normal controls, which is in agreement with the study of Segundo et al[8], but contrary to others. This conflict may be due to the fact that that several of these studies compared patients receiving CNI-based immunosuppression with those receiving RAPA. Our results (which include a healthy control group for comparison) suggest that RAPA has no deleterious effect on Treg numbers. However, the number of Tregs in patients treated with CNI appears to be reduced compared with healthy controls [16]. Therefore, Treg cell numbers in peripheral blood may not be a suitable predictor for transplant rejection.

It is known that Foxp3^+ ^nTregs are not only found in recipient lymphoid tissue after transplantation [17], but also at the graft site[18]. We hypothesized that lymphoid-tissue Tregs might be effective at blocking the initiation of an aggressive response against the graft, whereas at the graft site they may inhibit the activity of aggressive cells that have escaped regulation and migrated into the graft tissue[19]. Therefore, we examined the Foxp3 expression in both the allograft and lymphoid tissues using IHC and real-time PCR. We found no Foxp3 expression in the allograft hearts at the selected time point, but the expression of Foxp3 mRNA and protein increased significantly Day 1 post-transplant in the Con group.

We then examined the number of Tregs/CD4+ T cells in the peripheral blood, spleen, lymph nodes and thymus of healthy rats using flow cytometry. We found the greatest number of Tregs in the lymph nodes (3.66 ± 0.84%) followed by spleen (2.24% ± 1.28%), indicating that the lymph nodes are the major site of Tregs activity.

When a single dose of RAPA was given on Day 3 post transplantation, Foxp3 expression was increased. This suggests that lymphoid tissue might be the site where Tregs suppress activated effector T cells. While, when RAPA was given on Day 1 post transplantation, Fxop3 expression was decreased. This was conformity with Wang et al. 1992[1], Poston et al. 1999[2], Ikonen et al. 2000[3] and Dambrin et al.2003[4], which delayed treatment with RAPA effectively slows the progression of both allograft rejection. Furthermore, RAPA, in clinical treatment, is not traditionally used after transplantation at once.

The efficacy of RAPA in preventing acute rejection is known to be related to trough drug levels[20,21]. Therefore, we assessed the pharmacodynamics of RAPA using HPLC. We found RAPA was below its effective concentration (5-15 ng/ml) 5 days after treatment (Fig 2). Therefore, the second peak in Foxp3+ Treg cell numbers seen in the 3D group at Day 7 may reflect the normal alloresponse in the Lewis rat recipients. In addition, the last treatment time point should be in 5 days.

There are three general modes of suppression (cell contact-dependent suppression, the production of inhibitory cytokines or the consumption of limiting growth factors) that may explain the inhibitory actions of Tregs on activated T cells [22]. It has been proposed that the production of inhibitory cytokines[14,23] such as IL-10 and TGF-β1 play an important role in this suppression. However, measurements of cytokine concentrations in cell culture supernatants are typically very low, and picomolar concentrations are unlikely to trigger cellular responses. Therefore, biologically active cytokine concentrations may only be achieved in the microenvironments surrounding the secreting cells. Therefore, we examined the IL-10 and TGF-β1 mRNA expression by Tregs both in vivo and in vitro. When RAPA was given on Day 1 post transplantation, Foxp3 expression was decreased while IL-10 expression was increased. However, on Day 3 post transplantation both Foxp3 and TGF-β1 expression increased, but not that of IL-10 (Fig 6). These results support the suggestion that there is a role for cell-surface or secreted TGF-β1 in CD4^+^CD25^+ ^Treg-mediated suppression [24-28].

Interestingly, our data show the time of RAPA administration has differing effects upon Foxp3 expression and cytokine secretion by Tregs. As far as we are aware, this is the first time that this has been reported. We propose that RAPA administration on Day 1 post-transplant enhances iTreg proliferation, which then suppresses Teff cells by secreting IL-10, while RAPA administration on Day 3 post-transplant increases nTreg proliferation that in turn, inhibits Teff cells by secreting TGF-β1. The mechanisms responsible for these effects need further investigation.

## Conclusion

In conclusion, our data indicate that Foxp3+Tregs inhibit Teff cell activity within the peripheral lymphatic tissues by secreting cytokines such as TGF-β1. We also show that the number of circulating Tregs may not be a suitable predictor of transplant rejection.

## Authors' contributions

Juan Shan and Yuchuan Huang carried out the molecular genetic studies, participated in the sequence alignment and drafted the manuscript. Jie Zhang carried out the immunoassays. Chuntao Zhang participated in the sequence alignment. Youping Li and Yuchuan Huang participated in the design of the study and performed the statistical analysis. Li Feng and Shengfu Li conceived of the study, and participated in its design and coordination and helped to draft the manuscript. All authors read and approved the final manuscript.

## Supplementary Material

Additional file 1 **the histology of Foxp3 expression in spleens**. We assessed the dynamic changes in the number of Foxp3+ Tregs in the spleens by immunohistochemistry. Tissue sections were stained using an anti-Foxp3 monoclonal antibody. Con group POD 1: 1^st ^day after transplantation; POD 3: 3^rd ^days after transplantation; POD6: 6^th ^day after transplantation; 1 D group 1+1d: 1^st ^day after RAPA treatment in 1D group; 1+3d: 3^rd ^day after RAPA treatment in 1D group; 1+7d: 7^th ^day after RAPA treatment in 1D group; 1+10d: 10^th ^day after RAPA treatment in 1D group; 3D group 3+1d: 1^st ^day after RAPA treatment in 3D group; 3+3d: 3^rd ^day after RAPA treatment in 3D group; 3+7d: 7^th ^day after RAPA treatment in 3D group; 3+11d: 11^th ^day after RAPA treatment in 3D group. Click here for file

Additional file 2 **the histology of Foxp3 expression in lymph nodes (LN)**. We assessed the dynamic changes in the number of Foxp3+ Tregs in the lymph nodes(LN) by immunohistochemistry. Tissue sections were stained using an anti-Foxp3 monoclonal antibody. Con group POD 1: 1^st ^day after transplantation; POD 3: 3^rd ^days after transplantation; POD6: 6^th ^day after transplantation; 1 D group 1+1d: 1^st ^day after RAPA treatment in 1D group; 1+3d: 3^rd ^day after RAPA treatment in 1D group; 1+7d: 7^th ^day after RAPA treatment in 1D group; 1+10d: 10^th ^day after RAPA treatment in 1D group; 3D group 3+1d: 1^st ^day after RAPA treatment in 3D group; 3+3d: 3^rd ^day after RAPA treatment in 3D group; 3+7d: 7^th ^day after RAPA treatment in 3D group; 3+11d: 11^th ^day after RAPA treatment in 3D group. Click here for file

## References

[B1] Wang ME Stepkowski SM Ferraresso M Kahan BD Evidence that rapamycin rescue therapy delays rejection of major (MHC) plus minor (non-MHC) histoincompatible heart allografts in rats. Transplantation 1992 54 4 704 709 1412762 10.1097/00007890-199210000-00027

[B2] Poston RS Billingham M Hoyt EG Pollard J Shorthouse R Morris RE Robbins RC Rapamycin reverses chronic graft vascular disease in a novel cardiac allograft model Circulation 1999 100 67 74 10393683 10.1161/01.cir.100.1.67

[B3] Ikonen TS Gummert JF Hayase M Honda Y Hausen B Christians U Berry GJ Yock PG Morris RE Sirolimus (rapamycin) halts and reverses progression of allograft vascular disease in non-human primates Transplantation 2000 70 969 975 10.1097/00007890-200009270-00015 11014651

[B4] Dambrin C Klupp J Bîrsan T Luna J Suzuki T Lam T Stähr P Hausen B Christians U Fitzgerald P Berry G Morris R Sirolimus (Rapamycin) monotherapy prevents graft vascular disease in nonhuman primate recipients of orthotopic aortic allografts Circulation 2003 107 2369 2374 10.1161/01.CIR.0000065576.80196.A4 12719285

[B5] Gao W Lu Y El Essawy B Oukka M Kuchroo VK Strom TB Contrasting effects of cyclosporine and rapamycin in de novo generation of alloantigen-specific regulatory T cells Am J Transplant 2007 7 1722 1732 10.1111/j.1600-6143.2007.01842.x 17511761 PMC3831357

[B6] Wood KJ Sakaguchi S Regulatory T cells in transplantation tolerance Nature Review Immonology 2003 3 199 210 10.1038/nri1027 12658268

[B7] Demirkiran A Kok A Kwekkeboom J Kusters JG Metselaar HJ Tilanus HW van der Laan LJ Low circulating regulatory T-cell levels after acute rejection in liver transplantation Liver Transpl 2006 12 277 284 10.1002/lt.20612 16447185

[B8] Segundo DS Ruiz JC Izquierdo M Fernández-Fresnedo G Gómez-Alamillo C Merino R Benito MJ Cacho E Rodrigo E Palomar R López-Hoyos M Arias M Calcineurin inhibitors, but not rapamycin, reduce percentages of CD4+CD25+Foxp3+ regulatory T cells in renal transplant recipients Transplantation 2006 82 550 557 10.1097/01.tp.0000229473.95202.50 16926600

[B9] Battaglia M Stabilini A Roncarolo MG Rapamycin selectively expands CD4^+^CD25^+^FoxP3^+ ^regulatory T cells Blood 2005 105 4743 4748 10.1182/blood-2004-10-3932 15746082

[B10] Shan J Huang YC Feng L Ke NW Luo L Li CW Li YP A modified technique for heterotopic heart transplantation in rats Journal of Surgical Research in press 19691989 10.1016/j.jss.2009.05.024

[B11] Ono K Lindsey ES Improved technique of heart transplantation in rats J Thorac Cardiovasc Surg 1969 57 225 229 4884735

[B12] Streit F Christians U Schiebel HM Napoli KL Ernst L Linck A Kahan BD Sewing KF Sensitive and specific quantification of sirolimus (rapamycin) and its metabolites in blood of kidney graft recipients by HPLC/electrospray-mass spectrometry Clin Chem 1996 42 1417 1425 8787698

[B13] Roncarolo MG Gregori S Battaglia M Bacchetta R Fleischhauer K Levings MK Interleukin-10-secreting type 1 regulatory T cells in rodents and humans. Immunological Reviews 2006 212 28 50 16903904 10.1111/j.0105-2896.2006.00420.x

[B14] Schmidt-Lucke C Aicher A Romagnani P Gareis B Romagnani S Zeiher AM Dimmeler S Specific recruitment of CD4+CD25+ regulatory T cells into the allograft in heart transplant recipients Am J Physiol Heart Circ Physiol 2007 292 2425 2431 10.1152/ajpheart.01197.2006 17237241

[B15] Meloni F Cascina A Paschetto E Marone Bianco A Morosini M Pellegrini C Fietta A Vitulo P Pozzi E Viganò M Monocyte chemoattractant protein-1 levels in bronchoalveolar lavage fluid of lung transplanted patients treated with tacrolimus as rescue treatment for refractory acute rejection Transplant Proc 2003 35 1523 1526 10.1016/S0041-1345(03)00476-7 12826211

[B16] Baan CC van der Mast BJ Klepper M Differential effect of calcineurin inhibitors, anti-CD25 antibodies and rapamycin on the induction of FOXP3 in human T cells Transplantation 2005 80 110 10.1097/01.TP.0000164142.98167.4B 16003241

[B17] Hara M Kingsley CI Niimi M Read S Turvey SE Bushell AR Morris PJ Powrie F Wood KJ IL-10 is required for regulatory T cells to mediate tolerance to alloantigens in vivo J Immunol 2001 166 3789 3796 11238621 10.4049/jimmunol.166.6.3789

[B18] Graca L Cobbold SP Waldmann H Identification of regulatory T cells in tolerated allografts J Exp Med 2002 195 1641 1646 10.1084/jem.20012097 12070291 PMC2193557

[B19] Demirkiran A Kok A Kwekkeboom J Kusters JG Metselaar HJ Tilanus HW van der Laan LJ Low circulating regulatory T-cell levels after acute rejection in liver transplantation Liver Transpl 2006 12 277 284 10.1002/lt.20612 16447185

[B20] Fryer J Yatscoff RW Pascoe EA Thliveris J The relationship of blood concentrations of rapamycin and cyclosporine to suppression of allograft rejection in a rabbit heterotopic heart transplant model Transplantation 1993 55 340 345 10.1097/00007890-199302000-00021 8434385

[B21] Yakimets WJ Lakey JR Yatscoff RW Katyal D Ao Z Finegood DT Rajotte RV Kneteman NM Prolongation of canine pancreatic islet allograft survival with combined rapamycin and cyclosporine therapy at low doses: rapamycin efficacy is blood level related Transplantation 1993 56 1293 1298 10.1097/00007890-199312000-00001 8278991

[B22] Scheffold A Murphy KM Hofer T Competition for cytokines: Treg cells take all Nature Immunology 2007 8 1286 1287 10.1038/ni1207-1285 18026078

[B23] Maynard CL Harrington LE Janowski KM James RO Carlene LZ Alexander YR Casey TW Regulatory T cells expressing interleukin 10 development from Foxp3+ and Foxp3- precursor cells in the absence of interleukin 10 Nature Immunology 2007 8 931 941 10.1038/ni1504 17694059

[B24] Dieckmann D Bruett CH Ploettner H Lutz MB Schuler G Human CD4(+)CD25(+) regulatory, contact-dependent T cells induce interleukin 10-producing, contact-independent type 1-like regulatory T cells J Exp Med 2002 196 247 253 10.1084/jem.20020642 12119349 PMC2193931

[B25] Jonuleit H Schmitt E Stassen M Tuettenberg A Knop J Enk AH Identification and functional characterization of human CD4(+)CD25(+) T cells with regulatory properties isolated from peripheral blood J Exp Med 2001 193 1285 1294 10.1084/jem.193.11.1285 11390435 PMC2193380

[B26] Levings MK Sangregorio R Sartirana C Moschin AL Battaglia M Orban PC Roncarolo MG Human CD25+CD4+ T suppressor cell clones produce transforming growth factor beta, but not interleukin 10, and are distinct from type 1 T regulatory cells J Exp Med 2002 196 1335 1346 10.1084/jem.20021139 12438424 PMC2193983

[B27] Maloy KJ Salaun L Cahill R Dougan G Saunders NJ Powrie F CD4+CD25+ T(R) cells suppress innate immune pathology through cytokine-dependent mechanisms J Exp Med 2003 197 111 119 10.1084/jem.20021345 12515818 PMC2193798

[B28] Gorelik L Constant S Flavell RA Mechanism of transforming growth factor beta-induced inhibition of T helper type 1 differentiation J Exp Med 2002 195 1499 1505 10.1084/jem.20012076 12045248 PMC2193549

