# Supplementary figures and images for: Peripheral blood T Regulatory cell counts may not predict transplant rejection
Source: BMC Immunol. 2010 Jul 15;11:40. doi: 10.1186/1471-2172-11-40 (PMC2912834; doi:10.1186/1471-2172-11-40)

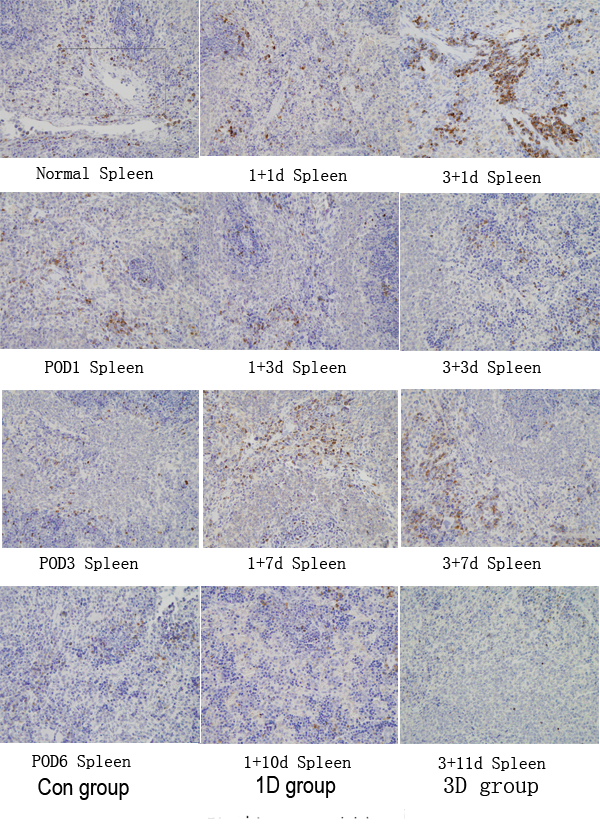

Supplement: Additional file 1 — the histology of Foxp3 expression in spleens. We assessed the dynamic changes in the number of Foxp3+ Tregs in the spleens by immunohistochemistry. Tissue sections were stained using an anti-Foxp3 monoclonal antibody. Con group POD 1: 1st day after transplantation; POD 3: 3rd days after transplantation; POD6: 6th day after transplantation; 1 D group 1+1d: 1st day after RAPA treatment in 1D group; 1+3d: 3rd day after RAPA treatment in 1D group; 1+7d: 7th day after RAPA treatment in 1D group; 1+10d: 10th day after RAPA treatment in 1D group; 3D group 3+1d: 1st day after RAPA treatment in 3D group; 3+3d: 3rd day after RAPA treatment in 3D group; 3+7d: 7th day after RAPA treatment in 3D group; 3+11d: 11th day after RAPA treatment in 3D group. [file 1471-2172-11-40-S1.TIFF]

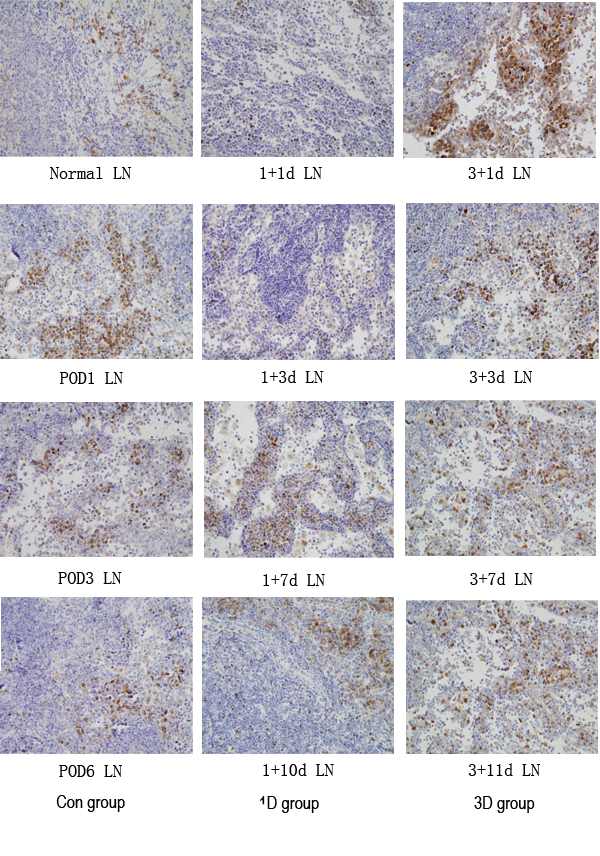

Supplement: Additional file 2 — the histology of Foxp3 expression in lymph nodes (LN). We assessed the dynamic changes in the number of Foxp3+ Tregs in the lymph nodes(LN) by immunohistochemistry. Tissue sections were stained using an anti-Foxp3 monoclonal antibody. Con group POD 1: 1st day after transplantation; POD 3: 3rd days after transplantation; POD6: 6th day after transplantation; 1 D group 1+1d: 1st day after RAPA treatment in 1D group; 1+3d: 3rd day after RAPA treatment in 1D group; 1+7d: 7th day after RAPA treatment in 1D group; 1+10d: 10th day after RAPA treatment in 1D group; 3D group 3+1d: 1st day after RAPA treatment in 3D group; 3+3d: 3rd day after RAPA treatment in 3D group; 3+7d: 7th day after RAPA treatment in 3D group; 3+11d: 11th day after RAPA treatment in 3D group. [file 1471-2172-11-40-S2.TIFF]
